# Cultural and co-designed principles for developing a Māori kaumātua housing village to address health and social wellbeing

**DOI:** 10.1186/s12889-024-18771-9

**Published:** 2024-05-15

**Authors:** John G. Oetzel, Corey Bragg, Yvonne Wilson, Rangimahora Reddy, Mary Louisa Simpson, Sophie Nock

**Affiliations:** 1 University of Waikato, Hamilton, New Zealand; 2 Te Rūnaka O Awarua, Bluff, New Zealand; 3 Te Rūnanga O Kirikiriroa, Hamilton, New Zealand; 4 Rauawaawa Kaumātua Charitable Trust, Hamilton, New Zealand

**Keywords:** Māori worldview, Housing, Participatory research, Health and wellbeing

## Abstract

**Background:**

The current study is a case study of a Māori (Indigenous people of New Zealand) organisation and their developmental processes in creating a kaumātua (older people) housing village for health and social wellbeing. This study identifies how a set of established co-design and culturally-centred principles were enacted when creating and developing the village.

**Method:**

A mixed-method concurrent design was used in creating the case with interviews (*n* = 4), focus groups (*N* = 4 with 16 total participants) and survey questionnaires (*n* = 56) involving kaumātua and organisation members.

**Results:**

Survey results illustrate that suitable and affordable housing are associated with self-rated health, loneliness, and life satisfaction. The primary purpose of the housing village was to enable kaumātua to be connected to the marae (community meeting house) as part of a larger vision of developing intergenerational housing around the marae to enhance wellbeing. Further, key themes around visioning, collaborative team and funding, leadership, fit-for-purpose design, and tenancy management were grounded in cultural elements using te ao Māori (Māori worldview).

**Conclusion:**

This case study illustrates several co-design and culturally-centred principles from a previously developed toolkit that supported the project. This case study demonstrates how one community enacted these principles to provide the ground for developing a housing project that meets the health and social wellbeing of kaumātua.

## Introduction

In Aotearoa New Zealand (Aotearoa), a housing crisis has developed spurred by colonial history, post-pandemic inflation, increased costs of housing (both rents and house prices), and increased demand for housing with limited housing stock [1–3]. While some of these features are recent, the colonial history has involved nearly two centuries of policies and practices that have resulted in loss of land, decreases in home ownership, and negative impacts to cultural identity and health/social wellbeing [1, 3–5]. The signing of the Treaty of Waitangi (Te Tiriti o Waitangi) as the founding of New Zealand brought a Western view of land ownership rather than the Māori view as kaitaki (guardians or stewards) of the land [1, 6]. This led to an alienation of Māori from the land which has negatively impacted on poverty and cultural identity (as Māori identify with the place they are from) [6]. Post World War II, many Māori moved to urban areas for jobs, but housing policies did not support the type of housing preferred (communal housing or papakāinga) and economic development did not transfer equally to Māori [1]. There was also loss of cultural practices through language policies in schools and practices that blamed Māori culture for social and economic inequities [7]. This historical trauma sets the foundation for the current housing crisis.

The housing crisis has affected kaumātua (older Māori, the Indigenous people of Aotearoa) hard for several reasons. Māori home ownership has dropped to less than 50% owning their homes in contrast to nearly 67% of other people from Aotearoa [5]. This is despite the fact that birth rates are increasing for Māori [8]. Further, retirement income provided by the government largely assumes home ownership and relatively low housing costs; it is a policy that does not take into account structural inequalities for Māori [9]. Low homeownership and limited income results in kaumātua living in social, temporary, or less than ideal housing situations [10–12]; in fact, Māori are over-represented as tenants or on waiting lists for social housing [13]. While rental homes may be another option, the limited income from retirement coupled with limited protection in rental agreements sometimes makes this a challenging option [4]. Overall, poor quality housing situations have negative impacts on health and wellbeing and kaumātua are more impacted in these negative outcomes than non-Māori in the same age group [11, 13].

Many Māori health and social service organisations and Iwi (tribe) organisations have sought to develop kaumātua and intergenerational housing villages (papakāinga) to address the housing crisis and meet health, wellbeing, and cultural needs of kaumātua. Kaumātua have key roles for Māori communities as “carriers of culture, anchors for families, models for lifestyle, bridges to the future, guardians of heritage, and role models for younger generations (p. 10)” [14]. Thus, kaumātua desire to live within a cultural life-space, and long-term residential care is not an appropriate option [15]. Further, kaumātua are at risk for social isolation that negatively impacts their health and social wellbeing [16–18], and thus there is a desire to have housing that can link them to their community and marae (community meeting house). These cultural, health, and social factors, along with a desire to ground housing and the developmental process in te ao Māori (Māori worldview), are foundational elements to developing these types of housing villages [19].

Simpson and colleagues [19] identified several principles for developing kaumātua housing. They conducted a retrospective interview study of kaumātua, visionaries, builders, and financiers who had developed a successful kaumātua housing village. Specifically, they identified nine key principles: a) create a clear shared vision and aspiration for kaumātua housing; b) create and maintain long-term high-trust collaborative relationships (with stakeholders in the housing sector); c) use strategic processes to benefit the housing project (e.g., project team meetings and monitoring systems); d) create and maintain a kaumātua community; e) support kaumātua mana motuhake (self-determination and independence) in the housing village; f) provide wrap-around support for kaumātua with multiple service providers to enhance wellbeing; g) offer culture-centred kaumātua community and housing; h) partner with kaumātua through co-design to meet their changing needs (e.g., have them review plans); and i) share experiences, learning and knowledge with other organisations to help them develop their own solutions. These principles were used to develop a toolkit to ensure the development of housing with a te ao Māori worldview [20].

The current study is a case study of a Māori organisation, Te Rūnaka o Awarua Charitable Trust (Awarua) in Bluff, New Zealand, and their developmental processes in creating a kaumātua housing village to enhance health and social wellbeing. Awarua followed the key principles and toolkit identified in previous research to guide their co-design and development process. This study identifies how the co-design and culturally-centred principles were enacted when creating and developing the Awarua kaumātua housing village.

## Method

The methodology is the He Pikinga Waiora (HPW; Enhancing Wellbeing) Implementation Framework [21], which utilises co-design, co-implementation and co-evaluation of research and implementation processes with communities and end users. HPW centres kaupapa Māori and emphasises self-determination and mātauranga (system of knowledge) Māori. Kaupapa Māori is a research approach that uses tikanga (cultural protocols) to promote Māori ways of being and normalising Māori worldviews, language, culture, and autonomy in research [22, 23]. Additionally, HPW is built on a strong international evidence base of four elements for best practice in co-design methodologies for research and practice: culture-centredness, community engagement, systems thinking, and integrated knowledge translation [21]. Culture-centredness suggests that effective system transformation happens when Indigenous cultural perspectives are part of defining problems and solutions [24]. Community engagement is collaborating with groups directly affected by a particular issue [25]. Systems thinking helps to address the complexity of the local contexts and the variety of determinants of housing problems [26, 27]. Finally, integrated knowledge translation emphasises co-design with end users in developing and implementing an intervention (e.g., housing village) [28, 29]. End users are the people who will use research findings and facilitate the translation from research to practice [30].

The framework was operationalised through a partnership involving university and community researchers. Three researchers from the University of Waikato along with one each from the Rauawaawa Kaumātua Charitable Trust and Te Rūnanga o Kirikiriroa formed the original research team; they were responsible for developing the original tool kit. This team partnered with three community researchers from Te Rūnaka o Awarua, which was building the housing village. These three researchers were stewarded by the Board of Trustees and collectively made decisions on research processes and direction. Six of the eight researchers involved are Māori; two are non-Māori but have worked in partnership with the original partners for more than a decade.

The current case study uses a concurrent, mixed-method case study [31]. Data collection included a survey questionnaire and interviews/focus groups. The partnership decided a multi-method approach was important to provide a rich picture of current housing perspectives/needs and the housing development process. The interviews and focus groups provided open-ended data from participant perspectives that are often viewed as consistent with Kaupapa Māori research. Surveys sometimes are viewed as inconsistent with Kaupapa Māori, but not if they are guided by Māori viewpoints and decision making. The research partnership wanted to have pre- and post-build data to make comparisons of the development. At the start of the project, the criteria for selecting tenants were not known. A survey allowed for many potential resident perspectives to be considered. Further, the partnership selected the questions and thought the information provided good context of the local system and complemented the information obtained in the interviews/focus groups.

## Case study

Bluff is the southern-most community in the South Island and includes 1,797 residents; 46.4% who are Māori including 12.6% 65 and over [32]. Home ownership (owned, partially owned or held in a family trust) is 75% which is much higher than New Zealand in general [32]. Since the early 2000s, Awarua has held a vision and aspiration to build papakāinga-type housing around Te Rau Aroha marae. Awarua decided that kaumātua housing was a manageable and appropriate first step in the larger housing vision. Despite the community's aspiration, there were some concerns from the Board about the project's viability. The original research team met with the Board, reviewed the toolkit and explained how they could "walk alongside" the community to support the build and research process. The Board was convinced the build was possible and accepted an invitation to participate in the research process. During the project’s first year, the Board completed architectural plans and a business case for the housing village that supported the project's financial viability. In the New Zealand Budget 2020, Cabinet agreed to provide a $3 billion investment in infrastructure to support New Zealand's economic recovery as part of the 11 May COVID-19 Response and Recovery Fund. The Government established the Infrastructure Reference Group to identify a pipeline of shovel-ready projects to support the economy during the COVID-19 rebuild. A proposal was prepared to build six kaumātua houses (prefabricated 50 m^2^ units) next to the marae. Awarua received $1.898 million for the project and provided an additional $300,000 of their own money.

## Data collection

Research ethics was provided by the University of Waikato’s Human Research Ethics Committee (WMS 20–54). Beyond traditional research ethics of informed consent, our study includes special consideration of research ethics with kaumātua and Māori organisations. First and foremost, we focused on kaumātua wellbeing and cultural safety. All data collection followed tikanga (cultural protocols) including karakia (prayer), mihi (acknowledgements), whakawhanaungatanga (making connections), and kai (food). We conducted openings and closings of meetings in Te Reo Māori (Māori language) to show respect for tikanga and conducted interviews and surveys in English to make sure all could participate (many in the community do not speak Te Reo Māori). Second, all data were the property of the participants and the organisation. The data were analysed and disseminated only after a collaborative approach to the analysis and with the approval of participants and the Board of Trustees.

Survey.

A survey questionnaire was administered to 56 kaumātua in December 2020, 22 pōua (older men; literally grandfather) and 34 tāua (older women; literally grandmother), with an average age of 73.42. The data was collected and compiled by the community researchers. The 14 questions asked about aspects of their current homes and wellbeing including self-reported health [33, 34], loneliness [35], life satisfaction [36], housing satisfaction and suitability [37] and neighbourhood satisfaction [38]. The primary purpose of the questionnaire was to provide an overall description of current housing and wellbeing indicators.

Interviews/focus groups.

The community researchers and university research team collaborated to conduct focus groups and interviews with kaumātua, visionaries, board members and project team members to get a sense of the community, current housing, and the building process. Visionaries are informal leaders who looked to move the project forward. A total of four focus groups and four interviews were conducted. The community researchers conducted three interviews with kaumātua that lasted an average of 40 min. One interview was with the project manager and conducted by one of the university researchers and lasted 30 min; this was the only interview or focus group conducted virtually. The university and community researchers conducted the focus groups lasting and average of 60 min. One of the focus groups included six kaumātua; a second included five visionaries; the final two focus groups were with six members of the Board of Trustees.

The interview and focus group protocols for kaumātua were similar. They explored topics such as current housing, perceptions of the neighbourhood, kaumātua needs around housing, desires for the kaumātua village, and insights for the leaders and the process. The visionaries' focus group centred around participant involvement, the inspiration and history of the vision, how the vision was communicated, and the relationships among key stakeholders. The focus group for the Board was organised around key principles in the toolkit to explore all phases of the proposed building process. This data collection occurred prior to the building of the village. The interview with the project manager took place during the building process and explored what was working well and not so well, along with key success factors and challenges. The interviews were transcribed and shared with the participants for feedback prior to data analysis.

## Data analysis

The quantitative data were analysed using descriptive statistics including bivariate correlations (establishes whether there is a statistical relationship among two items by comparing the variability of one with that of another). The qualitative data were analysed using the framework method [39]. The framework method uses an existing model to guide the initial coding of data and then uses inductive analysis (similar to thematic analysis) within the larger categories. The framework included eight chapters/principles of the toolkit [20]. A coding guide was created that involved identifying the people, focus, processes, and other aspects within each of the eight chapters. As three chapters focus on post-build components, the current framework and analysis centred on the first five chapters.

Two coders completed the analysis—a non-Māori university researcher and a Māori community researcher. We used an insider and outsider to strengthen the support for the findings. After coding the transcripts, the coders co-constructed a thematic analysis of the quotes within each of the key chapters. The themes were created through a comparative process and then validated by sharing with the community for feedback.

## Results

### Survey

We asked eight questions about the housing situation and three about overall wellbeing to provide a descriptive overview of the kaumātua. Most of the people live in a home they own (90%), with living in a home they rent (4%), someone else's home (4%) or an apartment (2%) as other situations. Table 1 displays the percentage of respondents who rated the current housing characteristics as high or low.

**Table 1:** Current housing situation and wellbeing indicators

**Question**	**Percent High**	**Percent Low**
How suitable or unsuitable do you think your home, house or flat is?	84% said their home is suitable or very suitable	7% said their home unsuitable or very unsuitable (9% were neutral)
In winter, is your home, house or flat colder than you would like?	50% said their home is not colder than they’d like	50% said their home is sometimes colder than they’d like; 20% said their home is often or always colder than they’d like
My house is free of damp and mould.	63% agreed or strongly agreed that their home is free of damp/mould	26% disagreed or strongly disagreed that their home is free of damp/mould (11% were neutral)
My house is adaptable enough to meet changes in my needs and circumstances.	70% agreed or strongly agreed that their house is adaptable	11% disagreed or strongly disagreed that their house is adaptable (19% were neutral)
When considering for all of the costs associated with my home, e.g., heating, electricity, etc., my housing situation is affordable.	62% agreed or strongly agreed that their home is affordable	13% disagreed or strongly disagreed that their home is affordable (25% were neutral)
My house is located in an area that allows me to be connected to my culture (e.g., marae, hapū or iwi).	83% agree or strongly agree that their house allows cultural connection	4% disagree or strongly disagree that their house allows cultural connection (13% were neutral)
I feel safe in my neighbourhood.	83% agree or strongly agree that they feel safe	6% disagree or strongly disagree that they feel safe (11% were neutral)
People who have contact with family and friends can still feel lonely sometimes, while those who have little contact may not feel lonely at all. In the last four weeks, how much of the time have you felt lonely?	85% report little or none of the time feeling lonely	15% report feeling lonely some or most of the time
Overall, how would you rate your health during the past 4 weeks?	77% report good, very good or excellent health	23% report fair or poor health
Please think about how you would rate your life overall with 0 = your worse possible life and 10 = to your best possible life. Where would you rate your life on this scale?	77% report life satisfaction as eight or higher	23% report life satisfaction as five, six or seven (none below five).

The previous table illustrates an overall high-quality housing situation and wellbeing indicators for most kaumātua including higher home ownership than census figures. Significant bivariate correlations (*p* < 0.05) with self-rated health included suitability of housing (*r* = 0.49) and affordability (*r* = 0.30). Suitability of housing was also significantly correlated with loneliness (*r* = -0.37) and life satisfaction (*r* = 0.63). Significant bivariate correlations with life satisfaction also included free of damp/mould (*r* = 0.29), affordability (*r* = 0.38), house enables connection to culture (*r* = 0.32) and safety (*r* = 0.42).

### Interviews/focus groups

The results are organised around five key elements within the toolkit framework. Within each element, we present the theme(s) and key quotes to support these themes. Most of the themes are framed from a cultural perspective of te ao Māori (worldview), with a specific focus on Ngāi Tahu whānui (tribe) living within Awarua.

#### Vision

The vision is about understanding the inspiration and aspiration for the housing community of Awarua. Although there is a larger housing crisis in Aotearoa generally, it is not as pronounced for kaumātua in Bluff as most own their own homes. Nonetheless, some of these homes are too large and do not meet mobility needs. While the Board of Trustees was interested in helping to meet these needs, the primary motivation for building the kaumātua village was creating housing near the marae (community meeting house) to create a connection to the community. This connection was not only for kaumātua but reflects the whole life course from birth to elders.

The original vision began in the early 2000s and was led by several kaumātua, particularly one tāua toa (strong older woman), which reflects the Awarua community (i.e., lots of leadership by women who also provide manaaki or care for the community). A focus group of younger visionaries who sought to move the vision to action noted,

*“From conception through to kaumātua and kuia (older men and women; local dialect uses pōua and tāua), and everything that our whānau (extended family) need in between, to thrive, and to be happy and healthy. That was the vision, and if the marae and the Rūnanga could be a platform for that. She (key visionary) was pretty much, ‘Let’s do it.’”*

A second person built off this idea:

*“Around that time, so this is coming 20 years ago; I think we were all sort of involved there, but (key visionary) was the main driver in that circumstance of really getting, in terms of the vision and that, along with others as well. So, we did a needs assessment up at the marae with it was mostly marae whānau, and a lot of us had just started having kids. So that was why the early childhood centre was the priority, and the first cab off the rank, so to speak.”*

The enactment of the vision began with building the early childhood centre right next to the marae and then the plan was developed to build the kaumātua flats. As one board member explained during a focus group:

*“Well, the vision was with (key visionary) and them because you know, ‘Get that built.’ Right, and they wanted it on the site actually. They wanted to have kaumātua flats, and I think they thought, and it wasn’t anything actually about housing and if we need to be safe, because people had homes. It was about, ‘well, we’re getting older and we want to be….When this gets cracking up here, and everything will be going; well, we might move in there.’ She said, ‘We might retire there.’ Yeah, become part of that community, in terms of what’s happening in the meeting house; all sorts of cultural stuff going on, and then we’ve got the babies over there. So, it was kind of that community inclusiveness, and that’s what that was about.”*

These quotes illustrate that connection to the marae was a priority for the kaumātua village.

As part of this vision, there was also a focus on building local capacity in the building process. Some saw this as a key secondary aim of the vision because having good employment was a way to help community members thrive. One of the younger visionaries noted the following about continuing to enact the vision:

*“The next part of the vision is papakāinga (intergenerational housing for a future development near the marae), and the new Ministers, and the new government, are hot on employment, skills development, redeployment, you know, Māori procurement; build your own with your own, etc.”*

Similarly, another person from the same group noted,

*"…and then working it and get skills, and not because you’re mates with this people, and they can come and work. It’s about building the capacity for our people."*

Thus, these quotes support the point about helping the community thrive to further support connection to the marae and the community.

#### Collaborative Team & Funding

The collaborative team includes the various partners and stakeholders who are involved in helping to make the vision a reality. Participants noted that there were several key partners who supported the due diligence process and the building process. The community met with the research team to explore a previous housing project and identify key learnings (both positive and negative experiences). Then, Te Puni Kōkiri—government policy advisor on Māori wellbeing and development (TPK)—provided seed funding and the Ministry for Business, Innovation, and Employment (MBIE), provided funding for the build. This resulted in them becoming collaborative partners with the Trust.

As the Board of Trustees was exploring the feasibility of the flats and they were exploring ways to keep costs down by using prefabricated building. One of the young visionaries noted,

*“We needed to find a kitset (prefab buildings) that was going to work, which was good cost, which everyone in the country’s looking for that… and then the TPK funds come along.”*

The TPK funds enabled Awarua to complete the feasibility and partner with a key person in a large project management company. The process identified that the flats were financially viable, which gave the Trust Board confidence to move forward. Another young visionary explained,

*“I don’t think it would have been possible because otherwise we would be just jumping around like we have been for so many years trying to dabble in and trying to make it move, but I think without the partnerships. It's the expertise I think that other people bring.”*

The community was ultimately able to obtain significant funding from the government to support the kaumātua flats. A national project manager was selected to oversee the construction process. Additionally, the contractor was based in a city about a 1.5-h plane ride away as they were the fabricators of the buildings. Thus, these entities became partners in the process. These further reflect the needs of having outside resources to support the community. As one board member offered,

*“We’ve got enough funding to do the build, and MBIE wanted a commitment, and was there a commitment from the Rūnanga. They wanted to hear that today, and we said, ‘Yes, we wish to commit this certain amount, 300k.’ … There’s another commitment for funding through TPK, for a project manager.”*

In addition, the partners for the kaumātua village, the community is considering the importance of relationships for the future housing plans. These relationships include transactional features such as access to resources and long-term features of trust-building and partnership. The latter features are reflective of te ao Māori, while the former are important functional elements. Ideally, they co-exist with the partners. One of the young visionaries offered insight into these relationships:

*“And these other local partners that are ready to kind of wrap-around us, and help move us into the next stage, especially with the Chamber of Commerce I think coming onboard and all the different housing people coming together. Because I don't think one entity or person or governance going to solve it. It's actually like, has to be a whole collaborative approach, and I think that's the key to it, but with the social outcomes I suppose.”*

Thus, relationships and partnership are key ways of working for the community to facilitate getting the building completed, but also ensure social and relational outcomes as well.

#### Leadership

Leadership reflects the direct oversight of the building process as well as the specific project management. For Awarua, the key theme was managing a dialectical tension of managing local versus outsiders. Key issues were the desire of the community to have local project management and a desire to have local contractors complete as much work as possible. One of the Board of Trustees noted,

*“That’s the reason why having a local project manager actually sits well. If your project’s going to be locally driven, and you’re going to be using local whānau, then it makes really good sense to have somebody on the ground that’s from here.”*

This was expanded by another board member considering contractors and partners:

*“We’ve got whānau; people that we would go to, to get the work, the mahi done. They would be our own whānau, and they’d be our own businesses and all that sort of that.”*

An additional board member further asserted,

*“We’ve still got an obligation through our funding contract with MBIE, to see if we get as many local people onboard as possible…It sounds like we’ve got to get up as much as we can, as high as 80 percent even which is really interesting. It would be Māori wouldn’t it if it’s MBIE; isn’t it for a Māori project?....and we’ve got those sorts of people around. We’ve got good Māori contractors.”*

While the desire was to have a lot of local contractors involved, the reality did not occur to this desired level. The local project manager explained,

*“It’s been really good working with [the larger project manager]. I have regular contact with [the lead]. He’s always taking phone calls, available for both Zoom and Teams meetings. However, it has been difficult with, our main contactor, as far as the contact and the communication that goes through [the project manager], but a lot of that is I’m contacting them directly as well; but it has been very challenging particularly with the procurement objectives, and prioritising of our local and Māori contractors.”*

The project manager further offered,

*“I guess when you’re working with a main contractor their end point is different to ours. Like relationships are really key. Maybe I was a bit naïve when I started the project, I was sort of reaching out to our builders and local businesses in our township. Who are our engineers that can do work with steel and metal? Who are the whānau that are landscapers? …But I guess at the end of the day when we’re working with a group that’s based in Christchurch [city north of Bluff, about a 1.5 hour flight], they’re looking for the best price, what the quote looks like, their timelines; but I think when you, you know, I contact these people and it’s like for one example, ‘it’s like, okay, you’re a builder, can I provide your name to our main contractor?’ They’ve got to give you a call and then often, it’s like there’s been no contact at all.”*

Thus, the nature of the three-way relationship was a barrier to getting local contractors involved and this was further exacerbated by the pandemic which limited direct face-to-face contact. Further, this experience encouraged Awarua to have close monitoring of processes and returning to those initial expectations to manage the process.

#### Fit for Purpose Design

The design of the kaumātua flats followed universal design standards and included a process of asking kaumātua their input on specific design features. The designs included some wish list elements but were also constrained by the amount of land available. More importantly, the emphasis on cultural elements were noted by participants.

Kaumātua offered some concerns with existing housing in expressing their wish lists. One of the concerns was having too large of a home as noted by one tāua,

*“Well, for me where I’ve got a big home there. My hubby died suddenly a couple of years ago. I’m left with a big home and family’s in Australia, oh, and Invercargill [city about 30 minutes north]. But I just though, it’s a big place to keep it up, your lawns. …But I do think that the little houses would be just ideal because then you’ve got no worries about getting your house fixed up if you have to, or whatever.”*

The preference for a smaller home was countered by another tāua,

*“I’m still working, for nothing; but I’m still working. I need an office or something that’s not impinging in my living room, you know, so that somewhere a moko (grandchildren) can stay because my whānau don’t live here.”*

A pōua noted another reason for some space:

*“But you need to have your memories. I find the European response to ageing and where older people should live to be very disrespectful of the mana (status) of those people, and I would hate to think that we follow that model.”*

The Board noted that there were constraints in land and hence they were only able to build six one-bedroom units. One board member explained their response to kaumātua needing space:

*“I’m just thinking about the one-bedroom unit and how we didn’t factor in… we didn’t think enough about that. That there will be ones that wanted a bit of extra space.…There just wasn’t enough room. I think there’s gonna be a compromise because this is the first lot. We might build some more with two [bedrooms] later on. I think we have to start… they did the measurements and that was the best we could get.”*

The Board decided to include individual storage sheds so that kaumātua would have space for their memories and material items.

Kaumātua also reflected needs to have cultural elements included—items that reflect a Māori feel as well supported cultural practices. One component was the desire for a shared space to have support communal activities:

*“I would think that a communal room or a hobby room or something like that would just about be where everybody can sort of meet in the one place, socialise, do hobbies, whatever. Yeah, because I guess the other part of it is that part of the reason for having several in one area is for mutual support.”*

The response was to create an outside communal space with sitting area to provide mutual support. As the units are near the marae, the decision was for an outside not an inside space since the marae is easily accessible to them. Further, carvings and other art were included on the property to provide a space that supports te ao Māori.

#### Tenancy Management Issues

The final theme revolved around tenancy issues post build. Participants discussed several issues from the selection process to affordability to wrap around services. These issues were ways to ensure the process was fair and to identify how to best support kaumātua.

Ensuring a transparent and fair process was one of the key concerns. One of the tāua explained,

*“I'm going back to the Māori here, and the kaumātua; it is really good to keep them informed and keep them in the loop. And if they are part of the process, it makes them feel as though they're having an input… I’m sorry to put emphasis on our Māori people but if you go back, like I’ve just told you two minutes ago, and you look at the housing in Bluff, every first or second house has got descendants of Ngāi Tahu living in it. And I think that is a thing that should happen, and if people feel as though they’re part of the process it makes it go much more smoothly.”*

Focus on fairness was also supported by a board member,

*“I suppose that depends on our criteria at the time of what, and it comes down to the criteria, and I suppose from my perspective, when you set a criteria, that has to be rock solid. You can’t say, ‘Well this is the criteria.’ And then someone, you know? You have to be, ‘This is it.’ And if they don’t meet it, you don’t meet it, and that you have to. Otherwise, you’re just going to be nowhere, and yes, just gonna piss everyone off.”*

Thus, various participants encouraged a clear and fair selection process that was transparent and informed by kaumātua (which was done through a survey process and a community hui or meeting). The kaumātua suggested that need and unsuitable housing should be the primary criteria, so these were weighted more heavily. Other criteria (e.g., connection to the marae, contribution to the community and being local) were directly shared with the community to ensure transparency.

A second tenancy management issue was supporting the financial constraints of kaumātua. Early in the process, the Board considered ways to make the flats affordable. One of the board members discussed funding models that would not work because they are not consistent with te ao Māori:

*“So you don’t fire like a [mainstream residential care provider] model and anything like that where people are asked to sell down their assets so they can come into these things? Shit no, no, no sorry. This is a Māori outfit.”*

As a result of this perspective, the Board decided to apply to become a Community Housing Provider to receive subsidised rents from the government to support kaumātua with needs to be able to afford the rents on their pensions. The government in New Zealand will pay part of the rent directly to a Community Housing Provider leaving kaumātua responsible for a smaller portion. These providers need to apply and meet certain requirements to qualify.

## Discussion

The purpose of this study was to explore how the co-design and cultural principles from an existing toolkit were enacted during the building process and decisions for a kaumātua housing village to address health and social wellbeing in Bluff, New Zealand. This study identifies key lessons learnt in the building process and around key cultural components.

The primary reason Awarua sought to build kaumātua housing is that it is part of a larger vision of connecting the community to the marae by developing housing and other programmes that serve the needs of whānau from birth to end of life. This housing village will enhance the health and social wellbeing of the community. While there is a housing crisis in most of Aotearoa, many of the kaumātua in the survey have suitable housing, own their own homes, and enjoy their neighbourhood. Further, the findings illustrate that suitable and affordable housing are key correlates of health and social outcomes (self-rated health, loneliness, and life satisfaction). This case illustrates the importance of developing a clear vision that meets the community's needs rather than just responding to the larger housing crisis [19]. In this case, Awarua want to provide kaumātua housing close to the marae to ensure the community can meet kaumātua needs and connect them to the marae to help reduce social isolation and ensure they age well with cultural connectivity [16–18].

Consistent with the extant literature on health and housing needs, Awarua considered various pragmatic issues to ensure affordable and suitable housing [4, 12, 19]. For example, they decided to develop one-bedroom flats given space and money constraints but also sought to make the rents affordable for kaumātua by becoming a Community Housing Provider. They also emphasised a te ao Māori worldview in making these pragmatic choices in decisions.

As a result of this worldview, a culture-centred perspective was integrated throughout the process. The design process is consistent with similar type of housing projects [19]. The design process involved participatory community design, which involves shared decision-making and communication [21]. It also involved building partnerships with key stakeholders in the housing sector, which enables the creation of age-friendly communities [40, 41].

A key implication of this study is the importance of using prior experience, history and even a toolkit to guide the research process. Awarua followed a culturally-centred toolkit for kaumātua housing [19]. The toolkit and experience of the research team served as useful frames of reference for Awarua to develop their project in a participatory/co-designed manner and to centre te ao Māori. It also enables the practicalities of financing, cross-sector collaboration, project management, tenancy management and wrap around services to help meet the health and social needs of kaumātua. While a toolkit cannot include every element, it did provide a useful reference to ensure a culturally-centred process that addressed key practicalities. However, not every aspect was realised with the difficulty of employing local contractors made difficult through the pandemic. The toolkit will need to be expanded to address some of these challenges and provide avenues for resolving such challenges. For example, the Awarua project manager suggests including a section on setting expectations before the building commences.

This study is not without limitations. The research process did not include participants from building or financing of the project, so those voices are absent. Further, as an in-depth case study, we cannot generalise to other housing projects even though there are lessons learnt that may enable other projects to thrive. In the future, research should be conducted to assess the impact of the housing village on the health and wellbeing of kaumātua and the larger community.

In conclusion, this case study illustrates several co-design and culturally-centred principles in developing a kaumātua housing project designed to meet health and social needs. The project demonstrates the importance of collaboratively developing a vision for a housing project and using that to guide the process. In this case, that vision is about developing kaumātua housing (and intergenerational housing in the future) to help maintain connections to the marae to reduce social isolation and enhance health. The study also illustrates the importance of using te ao Māori in designing the housing and making practical choices about developing and financing the project. Finally, this study offers important lessons for Indigenous community organisations who seek to develop kaumātua housing to meet the health and social wellbeing needs of their communities. As a postscript, the primary building was completed in 2022 with landscaping and other finer details taking until the end of 2022. The first residents moved into the village in 2023 and the organisation is still working on becoming a community housing provider.

## Data Availability

The datasets used and/or analysed during the current study are available from the corresponding author on reasonable request.

## Abbreviations

HPW: He Pikinga Waiora
MBIE: Ministry for Business, Innovation and Employment
TPK: Te Puni Kōkiri

## Glossary

*Aotearoa*: New Zealand
*Awarua*: Bluff harbour; abbreviation for the organisation in the study
*hāpū*: subtribe
*He Pikinga Waiora*: Enhancing wellbeing
*hui*: meeting
*iwi*: tribe
*kaumātua*: older people
*Kaupapa Māori*: research/services by Māori for Māori
*mana motuhake*: identity, autonomy, self-actualisation
*manaaki*: care
*Māori*: indigenous people of Aotearoa New Zealand
*Marae*: community meeting house
*mātauranga*: Māori system of knowledge
*Ngāi Tahu*: Iwi located in South Island of New Zealand
*papakāinga*: housing village (traditionally intergenerational)
*pōua*: older men; grandfather
*tāua*: older women; grandmother
*Te Ao Māori*: Māori worldview
*tikanga*: cultural protocols
*whānau*: closely connected kin group/extended family
